# Occurrence of *Alaria alata* in wild boars (*Sus scrofa*) in Poland and detection of genetic variability between isolates

**DOI:** 10.1007/s00436-020-06914-x

**Published:** 2020-10-26

**Authors:** Ewa Bilska-Zając, Gianluca Marucci, Anna Piróg-Komorowska, Małgorzata Cichocka, Mirosław Różycki, Jacek Karamon, Jacek Sroka, Aneta Bełcik, Iwona Mizak, Tomasz Cencek

**Affiliations:** 1 National Veterinary Research Institute in Puławy, Al. Partyzantów 57, Puławy, Poland; 2 Istituto Superiore di Sanità, Viale Regina Elena, 299 Rome, Italy; 3 Veterinary Hygiene Laboratory, ul. Brodowicza 13a, Krakow, Poland

**Keywords:** *Alaria alata*, DMS, AMT, Wild boars, Poland

## Abstract

*Alaria alata* is a trematode included among several emerging zoonotic parasites. The mesocercarial larval stage of *A. alata* named *Distomum musculorum suis* (DMS) may potentially be infective for humans. In the past, DMS was often observed in wild boar meat during the official *Trichinella* inspection by artificial digestion before a more specific and effective detection method, the *A. alata* mesocercariae migration technique (AMT), was introduced. In the present study, the AMT method was used to screen 3589 tissue samples collected from wild boars hunted in Poland during the 2015–2019 period. The survey mainly focused on the southern part of Poland with the majority of samples coming from Małopolskie, Świętokrzyskie, and Dolnoślaskie provinces; samples from ten additional provinces were also included. The total prevalence was 4.2% with mean abundance of 4.7 DMS. Occurrence was dependent upon environmental conditions (i.e., wetland habitats and water reservoirs) rather than on sex of the host or season in which they were hunted. The recovered trematodes were identified as *Alaria* spp. according to their morphological features. Molecular analysis of 18S rDNA and COI genes confirmed the species identification to be *A. alata* and documented genetic variability among the isolates.

**Electronic supplementary material:**

The online version of this article (10.1007/s00436-020-06914-x) contains supplementary material, which is available to authorized users.

## Introduction

*Alaria alata* (Diplostomidae, Trematoda), is a trematode discovered by Goeze in 1782. The mesocercarial stage of *A. alata* named *Distomum musculorum suis* (DMS) is the etiologic agent of a zoonosis known as alariosis. Currently, *A. alata* is considered an emerging zoonotic parasite. The life cycle of this trematode includes two intermediate hosts and various paratenic and definitive hosts. The first intermediate host is a freshwater snail (e.g., *Helisoma*, *Planorbis* spp.), which become infected by miracidia, the *A. alata* hatchling stage. The miracidia develop into sporocysts that produce cercaria, a fast-moving larval stage that emerges from its snail host and penetrates a tadpole and develops into a non-reproductive form, the mesocercaria. These mesocercariae can infect paratenic as well as canid definitive hosts (Möhl et al., 2009). Paratenic hosts such as snakes, frogs, rodents, and wild boars acquire *A. alata* infections by ingesting tadpoles or tissues of other infected paratenic hosts. Recent finding suggests that mesocercariae may infect humans, establishing them as an additional paratenic host (Mӧhl et al. 2009). To date, human cases have been attributed to frog legs and goose meat as sources of infection (Fernandes et al. 1976; Freeman et al. 1966; Kramer et al. 1996). Nevertheless, some researchers underline the need for a reassessment of the potential risk of wild boar meat as an origin of *A. alata* infection in humans (Hiromi González-Fuentes et al. 2014).

This parasite is generally considered to be non-pathogenic for the definitive host; however, large numbers of parasites may cause pulmonary hemorrhages during migration or enteritis when they mature in the small intestine. Within paratenic hosts, the mesocercariae can accumulate in various tissues leading to pathogenicity. *Alaria americana* is a closely related species which occurs in North America and is pathogenic to humans. It causes a variety of clinical syndromes ranging from low-grade respiratory disorders and skin lesions to diffuse unilateral subacute inflammations of the retina and optic nerve, and even anaphylactic shock with fatal consequences (Beaver et al. 1997; McDonald et al. 1994; Freeman et al. 1966; Fernandes et al. 1976). The lack of pathognomonic symptoms, atypical multiorgan changes, and the lack of eosinophilia (typical for other parasitic invasions), as well as the lack of specific serological tests makes alariosis difficult to diagnose (Wasiluk 2013).

*A. alata* is widespread in many environments because of the broad range of hosts including birds, amphibians, reptiles, and mammals (Riehn et al. 2012), and a number of studies have been conducted in Poland in order to understand *A. alata* distribution among a variety of hosts (Wójcik et al. 2002; Balicka-Ramisz et al. 2003; Szczęsna J 2008; Popiołek et al. 2003). A key role is played by aquatic fauna, especially snails, frogs, and tadpoles. Research conducted by Wójcik et al. (2002) showed the presence of *A. alata* in snails and frogs and indicated that the percentage of infected animals depended on the season. The prevalence in amphibians was highest in spring (100%) and then declined in autumn (~ 30%). The prevalence in intermediate hosts determines the frequency of infections in paratenic and definitive hosts. According to studies conducted in western Poland since 2003, the prevalence in foxes was 21.8% (Balicka-Ramisz et al. 2003). Further studies showed a prevalence of 2.2% in wolves (Popiołek et al. 2003) and a single case of infection in a Eurasian lynx (Szczęsna et al. 2008). The most recent investigation showed that the percentage of red foxes infected with *A. alata* varied significantly among regions (Karamon et al. 2018). In northern regions, the prevalence was very high (above 90%), while it was several times lower in southern areas (15.2% and 24.7% for southwestern and southeastern regions, respectively). Little information is available on the prevalence of *A. alata* in paratenic hosts in Poland; nevertheless, a wide range of wild fauna is believed to be involved in the maintenance of this parasite in the environment. Recently, DMS has been more frequently found in meat of wild boars during official *Trichinella* inspections using artificial digestion (Commission regulation (EC) no 2015/1375). A very recent study using mesocercariae migration technique (AMT) indicated that in some regions of Poland, prevalence of *A. alata* in wild boars exceeds 65% (Strokowska et al. 2020).

The majority of studies on mesocercariae species identification were based on morphological characters observed under a microscope. Molecular identification of *A. alata* has been very limited and concerned mainly with confirmation of morphological identification. Molecular data available in publications and GenBank database consist of a limited number of partial DNA sequences of internal transcribed spacer 2 (ITS2), 28S ribosomal RNA, and cytochrome C oxidase subunit I (COI). To our knowledge, there is no data on molecular characterization of *A. alata* occurring in Poland.

The aim of this study was to estimate the prevalence of *A. alata* in wild boar hunted in Poland and provide molecular characterization of infections when found. Using the AMT method, we examined over 3500 samples for the presence of this parasite and report the prevalence from multiple provinces in Poland. We examined the relationship between infection prevalence and environment, sex of host, and hunting season. Furthermore, we sequenced the nuclear ribosomal 18S gene and mitochondrial COI gene in order to investigate genetic subdivisions.

## Material and methods

### Samples

The samples for the study were collected from wild boars hunted in Poland from January 2015 to December 2019. The study was particularly focused on samples from Małopolskie province, where hunters regularly asked for *Alaria* testing; however, additional samples sent from other provinces were also included in the study. Tissue samples were collected by trained hunters from the diaphragm and from the pharynx area, in particular, muscle, connective, adipose, glandular, and lymphatic tissues. Samples were delivered to the Veterinary Hygiene Laboratory (ZHW) in Kraków, Poland, from 1 to 3 days after collection. The samples were stored in refrigerators at 4–8 °C and tested for the presence of *A. alata* within 1 day upon arrival.

### DMS detection method

The samples, consisting in 30–32 g of tissue collected from each wild boar, were analyzed by Veterinary Hygiene Laboratory (ZHW) in Kraków, Poland, by a modified version of AMT.

Tissue samples were chopped into 0.5 cm pieces using scissors and transferred to a plastic sieve housed in a plastic funnel connected with a rubber hose clamped at the end. The samples were completely covered with water at 46 °C–48 °C and incubated at room temperature for 90 min to allow the migration of parasites into the water. After incubation, 20 ml ± 2 ml of fluid was collected in measuring cylinder and then transferred to a counting basin and examined by a stereo microscope at 40–60 magnification. The trematode larvae were identified as DMS based on motility and morphological features according to Riehn et al. (2010). The observed DMS were counted and transferred to Eppendorf tubes, fixed with 96% ETOH, and provided to National Veterinary Research Institute in Puławy for species identification and molecular characterization of the parasites.

### Statistical analysis

Confidence intervals of the percentages of infected wild boars were calculated according to the method described by Newcombe (1998). Differences in the prevalence of infections among provinces were estimated by a Chi-square test with Bonferroni correction for multiple comparisons. To determine differences in the prevalence among sex and seasons, the Chi-square test was used.

The distribution of the quantitative variable “abundance” was tested by the Shapiro-Wilk test, and the normality of the data was rejected. Differences between multiple groups (sex; season; province) of abundance were determined by the Mann-Whitney *U* Test (sex; province) or the Kruskal-Wallis test (season). The statistical difference in the abundance was calculated only for two provinces (Małopolskie and Świętokrzyskie) because in the remaining provinces, the number of positive samples was too low to be taken into account.

All statistical analyses were performed using Statistica 9.1 PL software (StatSoft Corp.) The differences were considered statistically significant when *p* < 0.05.

### Molecular characterization

For species identification, a single DMS isolated from each sample was used. The DNA purification was carried out using the DNA IQ System (Promega, USA) according to ‘manufacturer’s protocol. For the molecular characterization, 18S rDNA and cytochrome C oxidase subunit I (COI) genes were used (Table 1). Specific primers to amplify a variable region of approximately 232 bp of the nuclear 18S rDNA gene were designed from *A. alata* 18S rDNA sequence deposited in GenBank (AY222091.1, Olson et al. 2003). Universal primers (Bowles and McManus 1994) were used to amplify a variable sequence of approximately 450 bp inside the mitochondrial COI gene.

**Table 1:** Primer sequences used for 18S rDNA and COI amplification

Primer name	Sequence (5′ → 3′)	Gene target	Fragment size (base pairs)	References
18sFor	GGTAACTCCAGCTCCAA	18S rDNA	~ 232	Developed in-house
18sRev	ACACCCGTTTAAAGGCA			
JB3	TTTTTTGGGCATCCTGAGGTTTAT	COI	~ 450	Bowles and McManus 1994
JB4.5	TAAAGAAAGAACATAATGAAAATG			

Each DNA locus was amplified by PCR according to the following cycling conditions: An initial denaturation step at 94 °C for 5 min was followed by 40 cycles with denaturation at 94 °C for 10 s, annealing at 54 °C (18S) or 48 °C (COI) for 30 s and extension at 72 °C for 1 min. A final extension step at 72 °C for 5 min was included.

PCR products were run on a 2% agarose gel with SimplySafe dye (Euryx, Polska) and visualized under UV light. PCR products were purified using ExoSAP (Affymetrix, UK) according to manufacturer’ instructions and sent to Genomed S.A. company for standard Sanger sequencing.

The forward and reverse sequences were analyzed and aligned using the Clustal algorithm included in the bioinformatics platform CLC Workbench 8.0.1 (Qiagen, Hilden, Germany) and consensus sequences compared with GenBank data by BLAST nucleotide algorithm to confirm the species identification.

Evolutionary analyses were conducted in MEGA X (Kumar et al. 2018), using the maximum likelihood method and Tamura-Nei model with 1000 bootstrap replication.

## Results

Trematode larvae were detected in 151 (4.2%) of the 3589 tested wild boars hunted during the 2015–2019 period. Samples were collected each month; however, the majority of them originated from wild boars hunted in winter (35.1%) and autumn (29.8%), with less from spring (19.9%) and summer (15.2%). The age of wild boars ranged from 8 months to approximately 2 years as assessed by hunters. *A. alata* was found in 76 male and 75 female wild boars. The infection prevalence ranged from a maximum of 60% in the Zachodniopomorskie province to a minimum of 0% in the Podkarpackie, Opolskie, Lubelskie, and Mazowieckie provinces (Table 2). A statistically significant difference in prevalence (*p* < 0.05) was observed between some provinces in pairwise comparisons (Table 2). No statistically significant differences were found in prevalence due to seasonality or sex (*p* > 0.05).

**Table 2:** DMS prevalence and abundance in wild boars in Polish provinces

	Province	No. of tested animals	No. of infected animals	Prevalence (%) [CI]	Abundance range [mean]
1	Małopolskie^*2, 10^	3126	109	3.5 [2.87–4.19]	1–18 [4.4]
2	Świętokrzyskie^*1, 3^	179	27	15.1 [10.18–21.18]	1–21 [6.5]
3	Dolnośląskie^*2, 10^	108	4	3.7 [1.02–9.21]	2–4 [3]
4	Śląskie^*2, 10^	58	3	5.2 [1.08–14.38]	2–3 [2.5]
5	Podkarpackie	30	0	0	–
6	Lubuskie^*10^	21	1	4.8 [0.12–23.82]	6
7	Łódzkie^*10^	19	1	5.3 [0.13–26.03]	2
8	Wielkopolskie	17	2	11.8 [1.46–36.44]	2–4 [3]
9	Opolskie	11	0	0	–
10	Zachodniopomorskie^*1, 3, 4, 6, 7,^	5	3	60 [14.66–94.73]	1–12 [4.7]
11	Lubelskie	7	0	0	–
12	Pomorskie	2	1	50 [1.26–98.24]	1
13	Mazowieckie	1	0	0	–
	Total	3589	151	4.2 [3.57–4.92]	4.7

*Provinces between which a statistically significant different prevalence has been observed

Confidence interval (CI) and mean are shown in brackets for prevalence and abundance respectively

After the asterisk (*), numbers of voivodeships were listed sequentially, in accordance with column 1 of Table 1, between which statistically significant differences in prevalence were observed

The abundance ranged from 1 to 21 DMS (mean 4.7). No statistically significant differences in abundance between sex (*p* > 0.05) and seasons (*p* > 0.05) were found; however, higher abundance was observed in Świętokrzyskie (average 6.5) than Małopolskie (average 4.4) province (*p* = 0.037).

Amplification of 18S rDNA and COI was successful and of expected size for all 151 DMS tested.

The alignment of the 18S rDNA sequences obtained from all the analyzed mesocercariae showed 100% of sequence identity demonstrating an absence of variability for this genetic marker. Moreover, the BLAST analysis of the obtained 18S rDNA sequence showed 100% of identity with *A. alata* 18S rDNA sequences already deposited in GenBank (accession numbers HM022226.1, HM022227.1, HM022228.1) confirming the species identification.

**Fig. 1:**
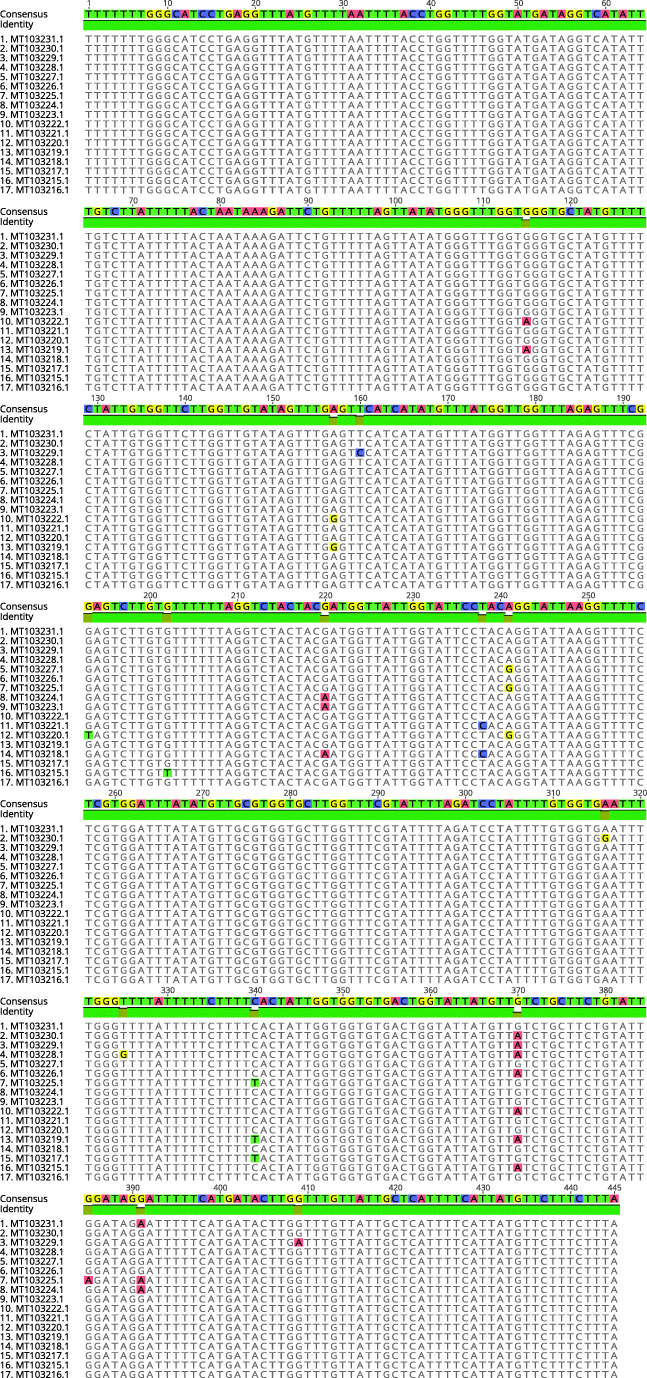
Alignment of the 17 different COI sequences of *A. alata* isolates collected from wild boars

**Fig. 2:**
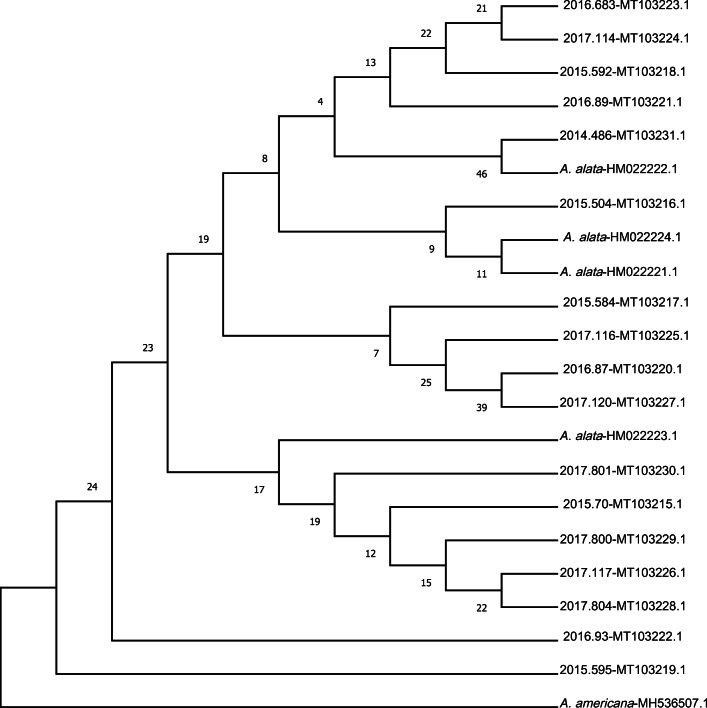
Unrooted cladogram of partial COI *A. alata* sequences together with *A. americana* (MH536507.1) as outgroup

At the COI locus, 17 unique genotypes (GenBank accession numbers MT103215–MT103231) were observed among 151 sequences, showing from 98.43% to 100% of identity among them (Supplementary Fig. S1) and differing from each other by one to seven single nucleotide variants (SNVs) (Fig. 1). The genotype frequency calculated over all the tested isolates ranged from 5 to 17. In the Małopolskie region, where the highest number of infected samples (72.8%) were collected, all 17 genotypes were present, with a frequency ranging from three to ten, suggesting the absence of a direct correlation between genotype and territory (Supplementary Fig. S2). The tree with the highest log likelihood is shown in Fig. 2 with the percentage of trees in which the associated taxa clustered together is shown next to the branches. The tree was built from the partial COI sequence alignment of the 17 genotypes, together with four *A. alata* sequences previously deposited in GenBank (HM022221.1–HM022224.1) and a sequence of *A. americana* (MH536507.1) added as outgroup. There were no significant subdivisions among the *A. alata* samples based on bootstrap support. Samples 2014.486 (MT103231.1) and 2015.504 (MT103216.1) showed 100% identity with GenBank COI sequences of *A. alata* isolated in wild boars in Latvia (HM022221.1 and HM022222.1 respectively).

## Discussion

**Fig. 3:**
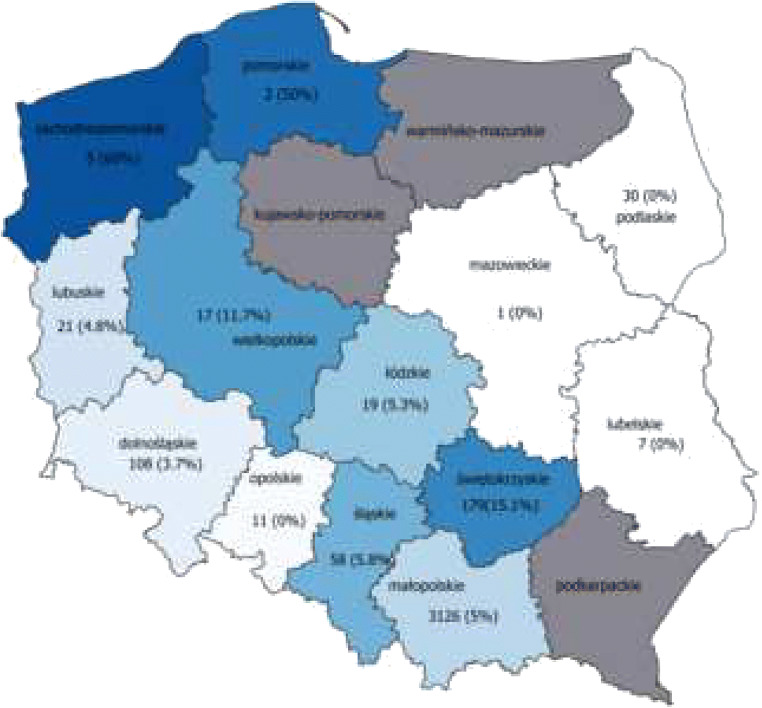
*Alaria alata* prevalence in wild boars in Poland. For each province, the number of tested wild boars and the parasite prevalence (in brackets) is reported. The rising of prevalence in provinces were signed by gradient blue color (from light blue to the dark blue); provinces from which no samples were tested was colored in gray

To our knowledge, only two studies of *A. alata* mesocercariae prevalence in wild boars using AMT in Poland have been done. The study of Renteria-Solis et al. (2018) showed only one infected wild boar (7.14%) over a total of fourteen animals hunted in Białowieża forest (Podlaskie province). A very recent study involving 221 wild boars hunted in five Polish provinces showed an *A. alata* prevalence ranging from 23.3 to 65.5% and an overall prevalence of 44.3% (Strokowska et al. 2020). The overall prevalence values shown in our study (4.2%) as well as the prevalence by province (from 0 to 60%) are very different from that reported by Strokowska et al. (2020). However, the area of sample collection in the two studies does not overlap, except in three provinces (Mazowieckie, Lubelskie, and Pomorskie) where the number of animals sampled in our study was extremely low (one to seven wild boars) (Fig. 3). Consequently, a direct comparison of results obtained in the two studies is not possible. Strokowska et al. (2020) emphasize that the regions from which their samples derive are rich in lakes, marshes, and rivers. This is an ideal habitat for intermediate hosts, such as water snails and frogs, as well as the wild boars. The present study focused wild boars mostly from Małopolskie, Świętokrzyskie, and Dolnoślaskie provinces which are characterized by a lower numbers of small water ponds and wetlands (https://stat.gov.pl, 2020.01.28). Therefore, the difference in prevalence between regions most likely reflects differences in the type of environment. This is supported by the lack of significant statistical differences between seasonality prevalence and sex prevalence in our and Strokowska et al. (2020) studies. In our opinion, the landscape composition is a significant point to take into consideration in a prevalence study on a parasite like *A. alata*, whose life cycle involves two water-associated hosts. It has been shown that *A. alata* is more prevalent in intermediate hosts in regions where wetlands are common (Wójcik et al. 2002). Florijančić et al. (2018) reported that the presence of mesocercariae was higher also in paratenic hosts, such as wild boars, living in plains that are periodically flooded. The presence of water reservoirs determines the occurrence of intermediate hosts (mainly planorbid snails) of *A. alata* which undoubtedly influences the prevalence of *A. alata* in paratenic (e.g., wild boars) and definitive hosts. The percentage of red foxes (definitive host) infected with *A. alata* vary significantly among regions in Poland with higher prevalence (above 90%) in the north and lower prevalence in southern areas (15.2% and 24.7% for southwestern and southeast regions, respectively) (Karamon et al. 2018). The findings of Karamon et al. (2018) were similar to a ten-fold difference in prevalence of *A. alata* in wild boars reported in the present study and Strokowska et al. (2020) in southeastern or north central regions of Poland, respectively. One other study determined that 57% of red foxes are infected by *A. alata* in central Poland (Borecka et al. 2009), which seems to correspond to our results of 15.1% of infected wild boars in Świetokrzyskie province which is in central of Poland.

The prevalence of *A. alata* in wild boars reported here is also similar to that reported by the other European countries: 11.5% of wild boar are infected in eastern Germany (Riehn et al. 2012), 6.8% in the Czech Republic (Paulsen et al. 2013), and between 0.6% to 12% in France (Portier et al. 2012). In our opinion, this is related to the scarcity of wetlands in the regions tested which these other authors also highlighted. Similar conclusions were made in studies of red foxes in Spain (Murphy et al. 2012), where *A. alata* was present in red foxes living in the well-irrigated valley of the Jarama river, but not in the desert regions of southern Spain.

The reliability of prevalence data is also related to the number of tested samples. Despite the high total number of samples analyzed, we cannot make a definitive conclusion about the occurrence of *A. alata* across all regions of the country, because of the focus on Malopolskie when collecting samples. Nevertheless, because of the number of samples tested and of the use of the gold standard method for DMS identification, this study represents the most comprehensive study on *A. alata* prevalence in wild boars carried out in Poland to date.

To our knowledge, this is the first study where such high numbers of DMS were subjected to molecular investigation. The molecular analyses of 18S rDNA and COI genes, initially done to confirm the morphological identification of the parasite, demonstrated noteworthy genetic variability between different isolates, indicating the occurrence of SNV differences between DMS collected from different wild boars. The phylogenetic analysis produced a dendrogram with low bootstrap values which suggests a lack of evolutionary differentiation among the *A. alaria* sampled. Alternatively, this may indicate a lack of informative sites given the relatively short DNA sequences collected. Further marker development and sequencing would be required to differentiate between these possibilities.

The identifications of seventeen different genotypes based on COI sequence analysis introduce a new area of investigation of *A. alata* as no intraspecific genetic differences had been previously reported in European *A. alata* isolates*.* This genetic variability may derive from several factors. The extent to which genetic variation is distributed among populations is determined by the interaction of various evolutionary forces, mainly genetic drift, selection, and migration. These evolutionary forces are affected by several biological and ecological factors, such as reproduction, breeding system, effective population size, and spreading capacity. The complex life cycle of *A. alata* that requires switching between intermediate, paratenic and definitive hosts could significantly favor gene flow within population (Lymbery and Thompson 2012). With higher gene flow, less regional differentiation is expected. Red foxes are one of the most frequently infected definitive hosts for *A. alata* in Poland. They have home ranges up to 25 km^2^ and their combined daily walking distance (up to 12 km) and occasional dispersal events play important roles for the spread of the species and have implications for various pathogen transmission (Walton et al. 2018). Such habits facilitate to spread the parasite to new areas and the transmission of different *A. alata* genotypes. This could explain the even distribution of the genotypes on the investigated regions. Because of a heteroxenous life cycle, *A. alata* has the ability to infect many animal species and one host may be likely infected with this parasite several times during its life. Therefore, it is possible that one wild boar or other intermediate or definitive host may be infected at the same time with different DMS genotypes. Unfortunately, in this study, only one DMS per samples was analyzed, making impossible to test this hypothesis. Further studies are required to understand both the population genetics of this parasite both within its host and across broader regions of Europe.

## Conclusions

In this work, samples were collected over five hunting seasons from 2015 to 2019 in Poland, allowing for a comprehensive study on the circulation of *A. alata* in wild boar, particularly in the southern provinces. The prevalence of this parasite in wild boars in southern provinces was found to be lower than that reported in recent studies involving northern provinces. The results support the hypothesis that *A. alata* occurrence depends on environmental conditions (i.e., wetland habitats and water reservoirs) rather than on sex of the host or time of year. Molecular investigation revealed intraspecific genetic variability between *A. alata* isolates; however, no correlation between specific genotypes and host geographical origin was observed.

According to the EU regulations, game meat that contains any parasitic pathogen potentially dangerous to humans should be treated as unfit for human consumption. However, the lack of obligation for routine inspection of wild boar meat by AMT method leads to an underestimation of DMS presence and puts consumers at risk. From the data shown here, it is recommended that government agencies implement a regular monitoring of this parasite in wild boar populations.

## Electronic supplementary material

Supplementary figure S1 Pairwise identity of partial COI sequences of *A alata* genotypes detected from wild boars in Poland. (JPG 1.58 mb)

Supplementary figure S2 Spatial distribution of COI genotypes of *A.alata* detected from wild boars in Poland. (PPTX 814 kb)
