# Supplementary figures and images for: Occurrence of Alaria alata in wild boars (Sus scrofa) in Poland and detection of genetic variability between isolates
Source: Parasitol Res. 2020 Oct 26;120(1):83–91. doi: 10.1007/s00436-020-06914-x (PMC7846538; doi:10.1007/s00436-020-06914-x)

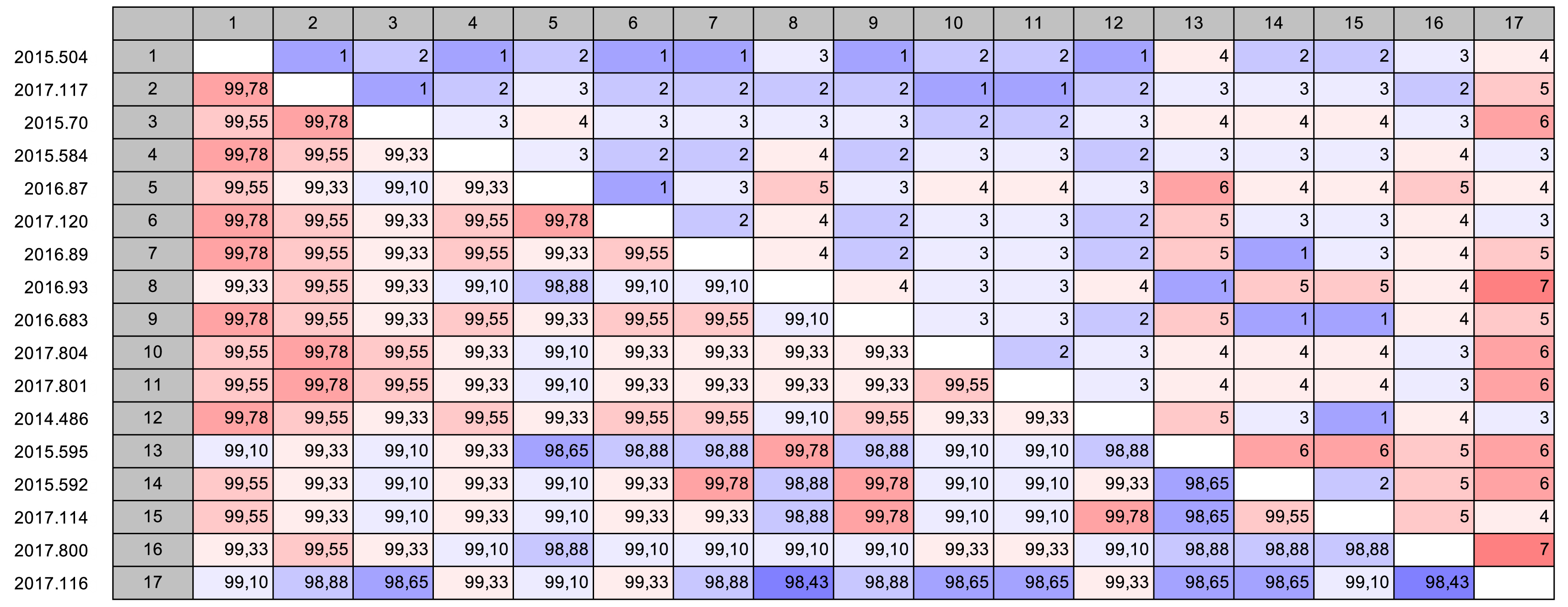

Supplement: Supplementary file 1 — Pairwise identity of partial COI sequences of A alata genotypes detected from wild boars in Poland. (JPG 1.58 mb) [file 436_2020_6914_MOESM1_ESM.jpg]
