# Supplementary material for: Occurrence of Alaria alata in wild boars (Sus scrofa) in Poland and detection of genetic variability between isolates
Source: Parasitol Res. 2020 Oct 26;120(1):83–91. doi: 10.1007/s00436-020-06914-x (PMC7846538; doi:10.1007/s00436-020-06914-x)
Supplement: Supplementary file 2 — Spatial distribution of COI genotypes of A.alata detected from wild boars in Poland. (PPTX 814 kb) [file 436_2020_6914_MOESM2_ESM.pptx]

## Slide 1
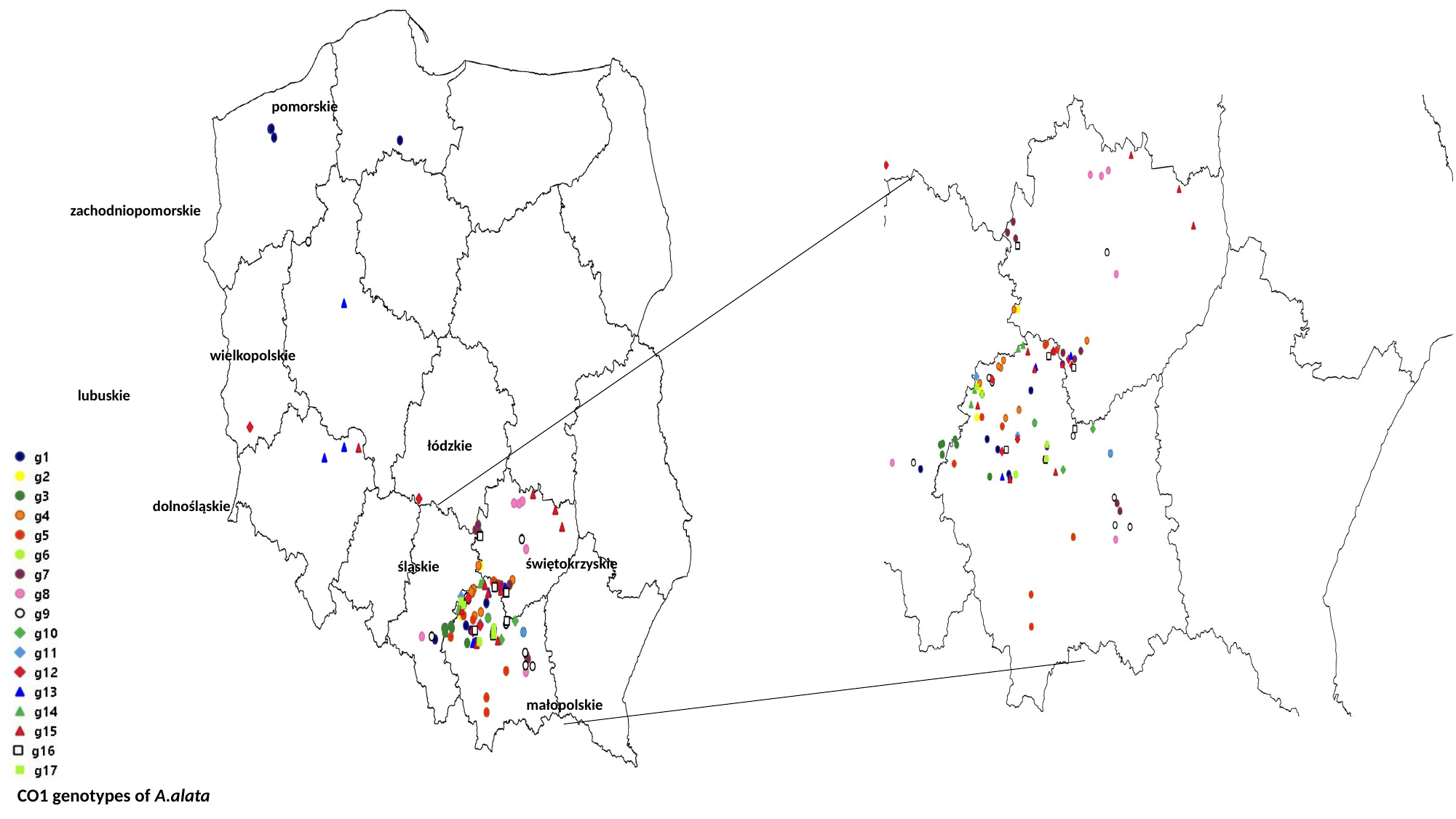

pomorskie
zachodniopomorskie
wielkopolskie
lubuskie
łódzkie
dolnośląskie
świętokrzyskie
śląskie
małopolskie
CO1 genotypes of A.alata
